# Modern Humans Did Not Admix with Neanderthals during Their Range Expansion into Europe

**DOI:** 10.1371/journal.pbio.0020421

**Published:** 2004-11-30

**Authors:** Mathias Currat, Laurent Excoffier

**Affiliations:** **1**Computational and Molecular Population Genetics Lab, Zoological Institute, University of BernBernSwitzerland; **2**Genetics and Biometry Laboratory, Department of Anthropology and Ecology, University of GenevaGenevaSwitzerland

## Abstract

The process by which the Neanderthals were replaced by modern humans between 42,000 and 30,000 before present is still intriguing. Although no Neanderthal mitochondrial DNA (mtDNA) lineage is found to date among several thousands of Europeans and in seven early modern Europeans, interbreeding rates as high as 25% could not be excluded between the two subspecies. In this study, we introduce a realistic model of the range expansion of early modern humans into Europe, and of their competition and potential admixture with local Neanderthals. Under this scenario, which explicitly models the dynamics of Neanderthals' replacement, we estimate that maximum interbreeding rates between the two populations should have been smaller than 0.1%. We indeed show that the absence of Neanderthal mtDNA sequences in Europe is compatible with at most 120 admixture events between the two populations despite a likely cohabitation time of more than 12,000 y. This extremely low number strongly suggests an almost complete sterility between Neanderthal females and modern human males, implying that the two populations were probably distinct biological species.

## Abstract 2

A model of human expansion into Europe reveals almost complete sterility between Neanderthal females and modern human males, implying that the two populations were probably distinct biological species

## Introduction

**Figure 1:**
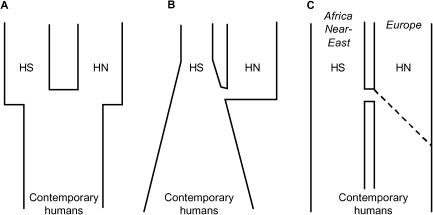
Different Models of the Interactions between Neanderthals and Modern Humans (A) Model of instantaneous mixing of unsubdivided Neanderthal and modern human populations. (B) Same as (A), but with an exponential growth of the modern human population having started before the admixture with Neanderthals. (C) Model of a progressive range expansion of modern humans into Europe. This model is spatially explicit, and the modern human population occupies a different range than the Neanderthal population before the admixture. Under this model, admixture is progressive and occurs because modern humans move into the territory of Neanderthals, a territory that shrinks with the advance of modern humans.

The “Neanderthals” or *Homo sapiens neanderthalensis* (HN) constitute a group of hominids, whose particular morphology developed in Europe during the last 350,000 y under the effect of selection and genetic drift, reaching its final form approximately 130,000 y ago (Klein 2003). This subgroup of hominids populated Europe and western Asia until the arrival of the first modern humans, *Homo sapiens sapiens* (HS), approximately 45,000 y ago (Mellars 1992). This arrival coincided with the beginning of Neanderthal decline, a process that occurred in less than 15,000 y and that is still not fully understood (Stringer and Davies 2001). An important question which remains to be assessed is whether Neanderthals could hybridize with modern humans and if they left some traces in the current modern human gene pool. While this hypothesis is excluded under the Recent African Origin Model (RAO), which postulates a complete replacement of former members of the genus by *H. sapiens,* it is central to the tenets of the multiregional hypothesis (Eckhardt et al. 1993; Wolpoff et al. 2000), which assumes a gradual transition from H. erectus to modern humans on different continents. From a paleontological and archaeological point of view the debate is still open, even if the supporters of the RAO (Stringer and Davies 2001; Rak et al. 2002; Schmitz et al. 2002) are gaining momentum over those supporting European regional continuity (Duarte et al. 1999; but see also Tattersall and Schwartz 1999). Recent morphological studies support a clear distinction between Neanderthals and modern humans (Harvati 2003; Ramirez Rozzi and Bermudez De Castro 2004), and genetic evidence, such as the clear divergence and monophyly of the HN mitochondrial DNA (mtDNA) control region (Krings et al. 1997, 1999; Ovchinnikov et al. 2000), suggested a long separation of the HN and HS female lineages (Krings et al. 2000; Scholz et al. 2000; Schmitz et al. 2002; Caramelli et al. 2003), with a divergence time estimated to lie between 300,000 and 750,000 y ago (Krings et al. 1997, 1999). The complete absence of Neanderthal mtDNA sequences in the current European gene pool, attested from the study of more than 4,000 recorded sequences (Richards et al. 1996; Handt et al. 1998) supported the absence of Neanderthal mtDNA leakage in the modern gene pool, but it was argued that even if some HN genes could have passed in the ancient Cro-Magnon gene pool, they could have been lost through genetic drift (Relethford 2001; Hagelberg 2003). Recently, several attempts were made at circumventing the drift problem by the direct sequencing of modern human fossils contemporary with the last Neanderthals. Cro-Magnon sequences were found very similar to those of current Europeans (Caramelli et al. 2003), even though contamination from modern DNA could not be completely excluded (Serre et al. 2004). All studies nevertheless agreed in showing the absence of Neanderthal sequence motifs among early modern human fossil DNA (Caramelli et al. 2003; Serre et al. 2004), but only Neanderthal contributions larger than 25% to the modern gene pool could be statistically excluded under a simple model (Figure 1A and 1B) of instantaneous mixing of Neanderthals and modern humans (Nordborg 1998; Serre et al. 2004). Thus, the problem of the genetic relationships between Neanderthals and modern humans remains fully open.

**Figure 2:**
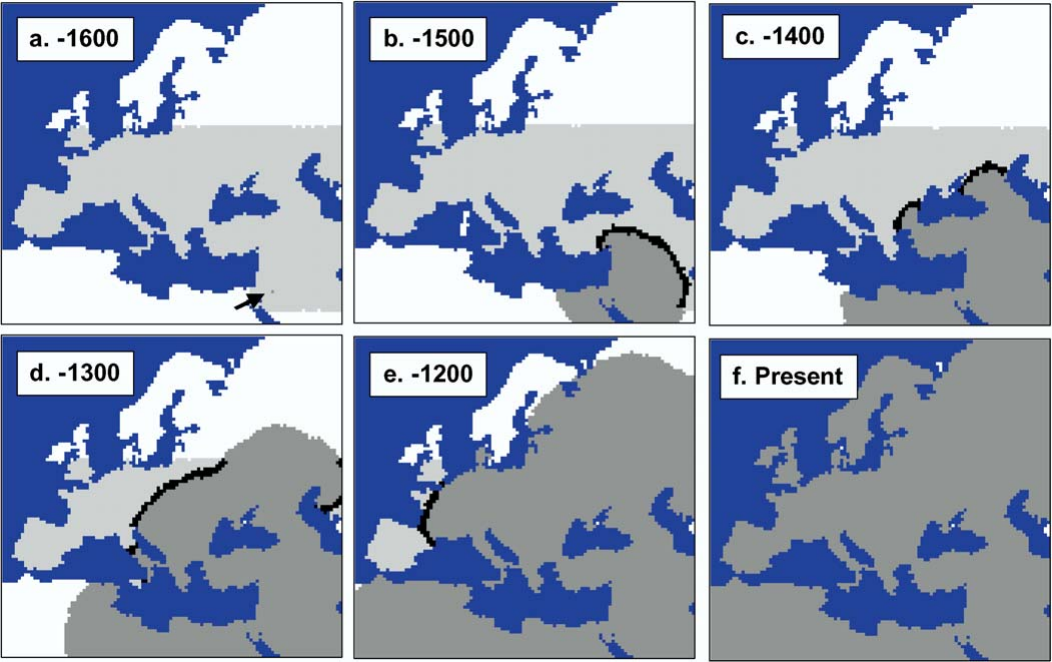
Range Expansion of Modern Humans into Europe from the Near East Simulations begin 1,600 generations ago, with the area of Europe already colonized by Neanderthals shown in light gray, and an origin of modern human expansion indicated by a black arrow (lane A). Lanes (B–F) show the progression of the wave of advance of modern humans (dark gray) into Europe at different times before present. The black band at the front of the expansion wave represents the restricted zone of cohabitation between modern humans and Neanderthals.

In order to further investigate this issue, we have developed a more realistic modeling of the admixture process between Neanderthals and early modern humans. In brief, the differences with previous approaches are the following (see Figure 1 and the Materials and Methods section for further details): (1) Europe is assumed to be subdivided into small territories potentially harboring two subpopulations (demes): an HN and an HS deme; (2) Europe is settled progressively by modern humans, resulting in a range expansion from the Near East. This range expansion implies also a demographic expansion of early modern Europeans, which stops when Europe is fully settled; (3) local population size is logistically regulated for both Neanderthals and modern humans; (4) we assume there is competition between modern humans and Neanderthals, resulting in the progressive replacement of Neanderthals by modern humans due to their higher carrying capacity caused by a better exploitation of local resources (Klein 2003); (5) Consequently, admixture between the two populations is also progressive and occurs in subdivisions occupied by both populations, in a narrow strip at the front of the spatially expanding modern human population (Figure 2); (6) coalescent simulations are used to estimate the likelihood of different rates of local admixture between modern humans and Neanderthals, given that Neanderthal mtDNA sequences are not observed in current Europeans.

The additional realism of this model makes it also more complex, and the range expansion and admixture processes will depend on several parameters, like the carrying capacities of the local populations, their intrinsic growth rate, the amount of gene flow between adjacent demes, the local rate of admixture between populations, or the geographical origin of the range expansion. Since it is difficult to explore this complex parameter space, we used archeological and paleodemographic information to calibrate the values of these parameters. For instance, the estimated duration of the replacement process (about 12,500 y, Bocquet-Appel and Demars 2000a) was used to adjust the speed of the expansion of modern humans and, thus, provided strong constraints on local growth and emigration rates. Based on available information, we thus defined a set of plausible parameter values considered as a basic scenario (scenario A). Local admixture rate, which is the parameter of interest here, was then varied, and its effect on the estimated contribution of Neanderthals to the current modern human gene pool was recorded. The sensitivity of admixture estimates to alternative parameterization of our model was studied in eight alternative scenarios (scenarios B to I), by varying each time the values of a few parameters.

## Results

### Expected Neanderthal Contribution to the Current European Gene Pool as a Function of Admixture Rates

The description of the nine envisioned scenarios for the colonization of Europe by modern humans is reported in Table 1. For each of these scenarios, the admixture rate, which is the parameter of interest in this study, was allowed to vary and only marginally influenced the cohabitation period and the replacement time of HN by HS (Table 1). Note that the cohabitation period at any given place (shown as a narrow black band on Figure 2) is limited to 6–37 generations, depending on the scenario.

**Table 1:**
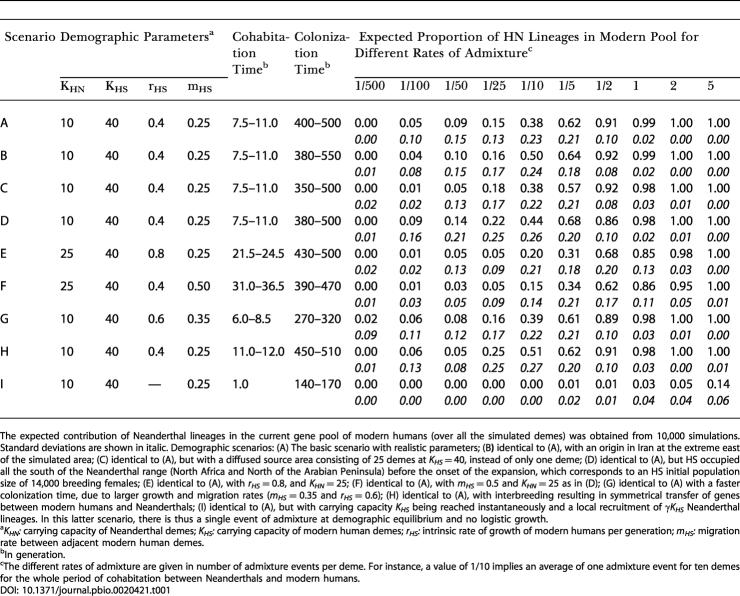
Expected Proportion of Neanderthal Lineages in the Present Modern Human Gene Pool under Different Demographic Scenarios

The expected contribution of Neanderthal lineages in the current gene pool of modern humans (over all the simulated demes) was obtained from 10,000 simulations. Standard deviations are shown in italic. Demographic scenarios: (A) The basic scenario with realistic parameters; (B**)** identical to (A), with an origin in Iran at the extreme east of the simulated area; (C) identical to (A), but with a diffused source area consisting of 25 demes at *K_HS_* = 40, instead of only one deme; (D) identical to (A), but HS occupied all the south of the Neanderthal range (North Africa and North of the Arabian Peninsula) before the onset of the expansion, which corresponds to an HS initial population size of 14,000 breeding females; (E) identical to (A), with *r_HS_* = 0.8, and *K_HN_* = 25; (F) identical to (A), with *m_HS_* = 0.5 and *K_HN_* = 25 as in (D); (G) identical to (A) with a faster colonization time, due to larger growth and migration rates (*m_HS_* = 0.35 and *r_HS_* = 0.6); (H) identical to (A), with interbreeding resulting in symmetrical transfer of genes between modern humans and Neanderthals; (I) identical to (A), but with carrying capacity *K_HS_* being reached instantaneously and a local recruitment of *γK_HS_* Neanderthal lineages. In this latter scenario, there is thus a single event of admixture at demographic equilibrium and no logistic growth

^a^ *K_HN_:* carrying capacity of Neanderthal demes; *K_HS_:* carrying capacity of modern human demes; *r_HS_:* intrinsic rate of growth of modern humans per generation; *m_HS_:* migration rate between adjacent modern human demes

^b^In generation

^c^The different rates of admixture are given in number of admixture events per deme. For instance, a value of 1/10 implies an average of one admixture event for ten demes for the whole period of cohabitation between Neanderthals and modern humans

**Figure 3:**
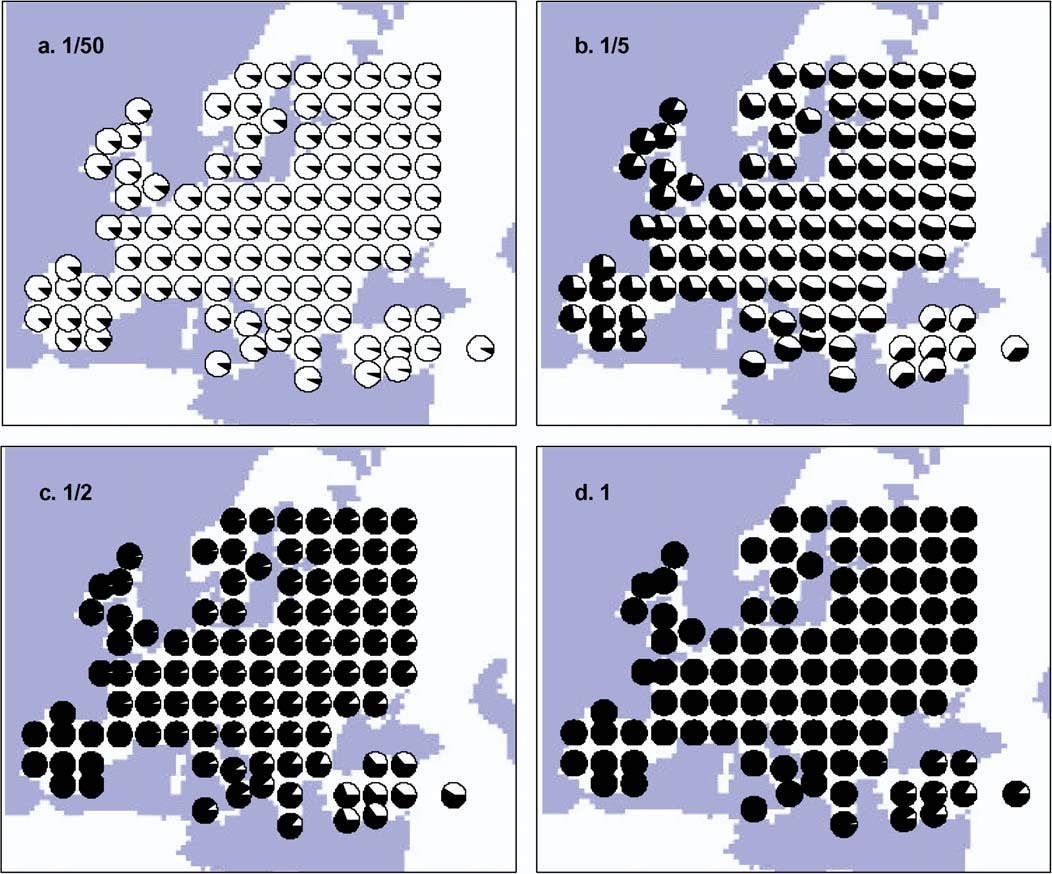
Expected Proportion of Neanderthal Lineages (in Black) among European Samples under Demographic Scenario A (Table 1) at Different Geographic Locations, for Different Interbreeding Rates (A) One admixture event on average per 50 demes over the whole period of cohabitation between Neanderthals and modern humans; (B) one admixture event per five demes; (C) one admixture event per two demes; (D) one admixture event per deme.

The expected proportion of Neanderthal genes in the gene pool of modern humans was estimated by coalescent simulations and is reported in Table 1 for different rates of admixture between Neanderthals and modern humans. At odds with previous estimates (Nordborg 1998 ; Gutierrez et al. 2002; Serre et al. 2004), our simulations show that even for very few admixture events, the contribution of the Neanderthal lineages in the current gene pool should be very large (see also Figure S1). For instance, in scenario A, with a 4-fold advantage in exploitation of local resources by modern humans, a single fertile admixture event in one deme out of ten over the whole period of coexistence between HN and HS should lead to the observation of 38% of HN genes in the present mtDNA HS gene pool (scenario A in Table 1). This proportion would be lower but still amount to 15% if the advantage of modern humans was reduced to 1.6 times over Neanderthals with the same admixture rate (scenario F in Table 1). With higher but still relatively low levels of admixture, a majority of Neanderthal genes should be expected in the current European gene pool (Table 1). For instance, with as much as two admixture events per cell over the total coexistence period of Neanderthals and modern humans, more than 95% of the current HS gene pool should be tracing back to Neanderthals, for all scenarios with logistic demographic regulation described in Table 1 (scenarios A to H). As shown on Figure 3, the proportion of current lineages that can be traced to Neanderthals is, however, not uniformly distributed over Europe in scenario of moderate or low interbreeding. A gradient should be visible from the source of the range expansion (which shows the largest proportion of modern human genes) toward the margins of the expansion (the British Isles and the Iberian Peninsula), which should then be expected to harbor a larger proportion of Neanderthal genes than the rest of Europe (Figure 3). However, this gradient would be relatively weak, and the expected proportion of HN lineages at any position is primarily affected by the degree of admixture between the two populations.

The finding that even minute amounts of interbreeding between Neanderthals and modern humans should lead to a massive introgression of Neanderthals' mtDNAs into the Cro-Magnon gene pool is somehow counterintuitive and deserves further explanations. The successful introgression of Neanderthal mtDNAs is due to a massive dilution of the modern human mtDNA gene pool into that of the pre-existing population (Chikhi et al. 2002) and to a low probability of being lost by drift at the time of introgression (see below). The dilution process can be seen as follows: An HN gene entering the HS gene pool at an early stage of the colonization process will lower the frequency of HS genes in the HS deme; the migrants sent from this deme to colonize an adjacent new territory can themselves harbor HN genes, so that a further HS deme can be founded by a mixture of HS and HN genes; additional admixture events will further lower the proportion of HS genes in HS demes. The repetition of these admixture and migration steps will thus rapidly dilute HS genes. Under this process, the European HS population can be fully introgressed by HN genes under scenarios A to H, if two or more admixture events occurred in each deme (see Table 1, last two columns). For such large rates of admixture, the fraction of HS genes in demes adjacent to the source of HS expansion is already diluted by more than 28% with HN genes (results not shown). Therefore, in the absence of counteracting selective forces, the dilution process repeated over several demes would hinder the spread of HS genes away from the source of the colonization. The range expansion would thus be mainly carried out by individuals having HN genes, explaining why the HS European population would appear fully introgressed by HN genes. The success of introgressing HN genes is also due to their integration into the HS deme while it is in a period of demographic (logistic) growth (see Figure S2), so that these introgressing genes are unlikely to be lost by genetic drift, and will, rather, be amplified by the logistic growth process occurring in the HS deme. In order to assess the importance of the period of logistic growth relative to the dilution process, we have modeled a range expansion process where a newly founded deme reaches instantaneously its carrying capacity, and where a given proportion of genes is recruited from the local Neanderthal gene pool. The results of those simulations (reported in Table 1 as scenario I) show that without logistic growth much larger interbreeding rates would be necessary to have the same impact on current human diversity. Indeed, the occurrence of two admixture events per deme over the whole cohabitation period would only lead to 5% of the current gene pool being of Neanderthal ancestry, instead of 100% when logistic growth is implemented.

### Estimation of Admixture Rates between Neanderthals and Modern Humans

**Figure 4:**
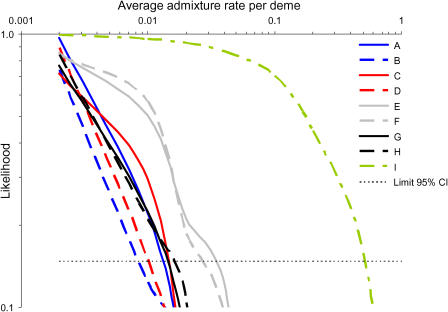
Likelihood of Different Rates of Interbreeding under the Nine Scenarios Described in Table 1 The horizontal bold dashed line corresponds to 14.7% of the maximum likelihood, defining the upper limit of a 95% confidence interval for the interbreeding rates (see, e.g., Kalbfleisch 1985).

The present results show that if Neanderthals could freely breed with modern humans, having progressively invaded their territory, their contribution to our gene pool would be immense. Since no Neanderthal mtDNA sequence has been observed so far among present Europeans, it is of interest to estimate the maximum admixture rate between Neanderthals and modern humans that would be compatible with an absence of Neanderthal genes, accounting for the current sampling effort and genetic drift over the last 30,000 y. This assessment was done by coalescent simulations. The likelihoods of different admixture rates are reported in Figure 4 for each scenario. Maximum-likelihood estimates are obviously obtained for a total absence of interbreeding between HS and HN, but here the interest lies in the upper limit of a 95% confidence interval. We see that the scenarios A to H can be divided into three groups. Scenarios A, C, G, and H lead to very similar upper bounds for the estimation of the maximum admixture rate (approximately 0.015 admixture events per deme; see Table 2). Similarity of results obtained for scenarios A and C show that the fact that the origin of the spread of modern humans was diffused over a large area or concentrated at a single point does not substantially influence our results. A shorter duration of the colonization of Europe by HS (approximately 8,000 y; scenario G) leads to an estimation very similar to that obtained under scenario A. Also the implementation of fully symmetric interbreeding between HN and HS (scenario H) leads to results almost identical to those obtained when we only allow breeding between HN females and HS males (scenario A). The place of origin for modern humans seems more important, as a putative origin in Iran (scenario B) or in North Africa (scenario D) leads to even lower maximum interbreeding rates (approximately 0.01 admixture events per deme) than if the source is located closer to Europe as in scenario A. Moreover, scenario D also shows that a much larger initial size of the HS population (14,000 breeding females instead of 40 in scenario A) does not reduce the final Neanderthal contribution to the HS gene pool. This is because we model local (at the deme level) and not global contacts between the two populations. Finally, scenarios E and F, corresponding to larger carrying capacities of Neanderthals, would be compatible with a larger amount of admixture between the two species (approximately 0.03 admixture events per deme), which is understandable given the longer cohabitation times under these scenarios (21–37 generations) than under scenarios A–D and G–H (6–12 generations). The estimates of the average number of admixture events per deme can be translated into a maximum number of interbreeding events having occurred over all Europe during the whole replacement process of Neanderthals by modern humans, as reported in Table 2. We find that, depending on the scenario, these maximum estimates range between 34 (scenario B) and 120 (scenario E) admixture events over the whole of Europe , which are extremely low values given the fact that the two populations have certainly coexisted for more than 12,000 y in that region.

**Table 2:**
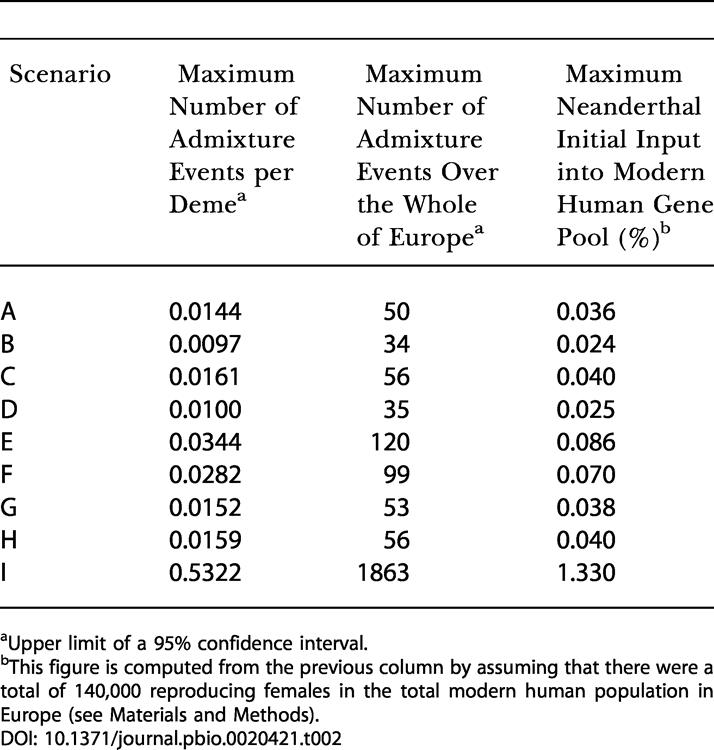
Measure of Genetic Interaction between Neanderthals and Modern Humans

^a^Upper limit of a 95% confidence interval

^b^This figure is computed from the previous column by assuming that there were a total of 140,000 reproducing females in the total modern human population in Europe (see Materials and Methods)

## Discussion

Our simulations show that the mitochondrial evidence in favor of no, or very little, interbreeding between Neanderthals and modern humans is much stronger than previously realized (Wall 2000; Nordborg 2001). We indeed find that the current absence of Neanderthal mtDNA genes is compatible with a maximum admixture rate about 400 times smaller than that previously estimated (Nordborg 1998; Serre et al. 2004). This initial estimate (25%) was, however, based on a simple but unrealistic model of evolution, assuming no population subdivision, constant population size, and a single and instantaneous admixture event between Neanderthals and modern humans. Taking into account the progressive nature of the range expansion of modern humans into Europe, the *maximum* initial input of Neanderthal genes into the Paleolithic European population can thus be estimated to lie between only 0.02% (scenario B) and 0.09% (scenario E) (Table 2). Our simulations of alternative scenarios of HS range expansion into Europe suggest that our results are not very sensitive to local HS growth rates, level of gene flow between neighboring HS demes, or the geographical origin of HS range expansion. It is also worth emphasizing that the final HN contribution to the European gene pool does not really depend on the size and spread of the population at the source of the range expansion (compare scenario A to C and D in Table 1). This is logical since the colonization process starts from a restricted number of demes at the edge of the pre-existing range in our model of subdivided population (see Figure 1C). If this model is correct, it implies that the current European genes should have coalesced in a small number of individuals present in the demes at the source of the colonization of Europe, or, in other words, that there was a bottleneck having preceded the range expansion into Europe. Available data on European mtDNA diversity indeed support this view, since most European populations do present a signal of Paleolithic demographic expansion from a small population, which could be dated to about 40,000 y ago (Excoffier and Schneider 1999).

Additional complexities of the simulation model could have been envisioned, like the possibility for long-range dispersal, some heterogeneity of the environment leading to different carrying capacities and preferential colonization routes, or uneven migration rates. However, these extra parameters would have been very difficult to calibrate due to the scarcity of paleodemographic data. Moreover, it is likely that they would not have lead to qualitatively different results. For instance, since long-range dispersal speeds up the colonization process (Nichols and Hewitt 1994), short range migration rates would need to be reduced, in order to preserve a realistic colonization time. But this reduction would have no effect on local cohabitation time, which is the important factor affecting admixture rates (Table 2). Another source of realism could be the implementation of a recent Neolithic expansion wave on top of a Paleolithic substrate. This additional expansion wave has not been implemented here, as it is clearly beyond the scope of the present study. However, our present results suggest that small amounts of admixture between the Paleolithic and the Neolithic populations would lead to a massive contribution of Paleolithic lineages among the current Europeans. This point is important as it implies that if Neanderthal lineages had been present among the Paleolithic populations, they would not have been erased by the spread of the Neolithic in Europe. If we were using previous estimations of the Neolithic contribution to the current European genetic pool of about 50% (Barbujani and Dupanloup 2002; Chikhi 2002), the effect of a Neolithic expansion would require our estimates of the initial input of HN into the modern pool to be roughly multiplied by two, but still be very small (0.07% for scenario A). Note also that the simulation of a pure acculturation process, which amounts to increasing the carrying capacity of populations after the Neolithic by a factor 250 has virtually no effect on the expected proportion of Neanderthal genes in current Europeans (see Figure S1). Another argument against a major influence of the Neolithic expansion stems from mtDNA studies, since the demographic expansion inferred from mtDNA diversity and dated to about 40,000 y ago (Excoffier and Schneider 1999) implies that most of the mtDNA lineages of current Europeans result from a Paleolithic range expansion (Ray et al. 2003). If the expansion of Neolithic settlers had fully erased Paleolithic mtDNA diversity, one would indeed not expect to see this Paleolithic expansion signal. It thus argues in favor of a minor contribution of Neolithic genes to the current European gene pool, as expected under our model of progressive range expansion with continuous mixing.

Compared to previous models assuming an instantaneous mixing of HN and HS populations (Nordborg 1998; Serre et al. 2004) (see Figure 1A), we find that extremely small Neanderthal contributions should still be visible in the European gene pool. It implies that HN genes have a much larger probability of persisting when entering a progressively invading HS population than when entering a stationary population. This is because HN genes enter the HS population in demes that are still growing in size (see Figure S2), which prevents them from being lost by genetic drift and which amplifies their absolute number in the deme, making it likely they will persist and reach observable frequencies in the global population. This process is actually similar to that occurring in an unsubdivided growing population (e.g., Otto and Whitlock 1997). Actually, if HN genes were to directly enter an unsubdivided HS population that grew exponentially until today (see Figure 1B), the current absence of HN genes would also imply a very small amount of Neanderthal introgression into our gene pool (Nordborg 1998; Serre et al. 2004). However, this continuous and global exponential growth process appears difficult to justify (Serre et al. 2004) and does not really apply to the late Pleistocene human population (Weiss 1984; Biraben 2003).

Under our model, the progressive range expansion (Figure 1C) and the local logistic growth contribute to reduce the probability of losing introgressed HN genes. Without logistic growth, much larger interbreeding rates would be necessary to have the same impact on current human diversity (see scenario I in Table 1 and in Figure 4). Under this scenario, the absence of Neanderthal mtDNA sequences in present Europeans is still compatible with a maximum of about 1,850 fertile breedings between Neanderthal females and Cro-Magnon males, corresponding to a maximum initial input of 1.2% Neanderthal genes into the European Cro-Magnon population (Table 2). This figure being 20 times larger than when assuming an initial logistic growth of newly founded populations, it shows that the local logistic growth and the progressive range expansion contribute equally to reducing the inferred admixture rate compared to the simple model assuming a single admixture event and an instantaneous settlement of Europe by modern humans (see Figure 1A) (Serre et al. 2004). However, because new territories are often colonized by a few migrants and not by whole populations, local logistic growth has been incorporated into most models of range expansion (e.g., Fisher 1937; Skellam 1951; Shigesada and Kawasaki 1997). It should thus be considered as a normal feature of range expansions.

Another important result of this study is to show that an expanding population or species is likely to have its own genome invaded by that of the invaded population if interbreeding is possible and gradual, which could explain some documented cases of mtDNA introgression (e.g., Bernatchez et al. 1995; Shaw 2002). Our results indeed suggest that introgression should occur preferentially in species having gone through a range expansion, and that the introgressing genome would be that of the invaded population and not that of the invasive species. Of course this result should only apply to the part of the genome that is not under selection or that is not linked to the selective advantage of the invaders. If the mitochondrial genome of modern humans was involved in their higher fitness, the absence of observed mtDNA introgression would not necessarily be due to an absence of interbreeding, but would rather result from an active selection process against crosses between Neanderthal females and modern human males, and one would therefore expect to see potential leakage of Neanderthal genes in our nuclear genome. While some evidence for the differential fitness of some mtDNA human genomes in distinct climates has been recently found (Mishmar et al. 2003; Ruiz-Pesini et al. 2004), it is unlikely that such differences were involved in the selective advantage of modern humans over Neanderthals. It is indeed doubtful that modern humans coming from the Middle East would have had mitochondria better adapted to the colder environment of Europe than Neanderthals, who had spent tens of thousands of years in such a climate (Tattersall and Schwartz 1999; Klein 2003). It is therefore more likely that modern humans' higher technology and higher cognitive abilities (Klein 2003), resulting in better resource processing and environmental exploitation, have allowed them to out-compete Neanderthals, and that mtDNA was selectively neutral in that respect. It should however be kept in mind that our conclusions assume no sex bias in interbreeding rates. Studies of fossil Y chromosome or nuclear DNA would be needed to examine the basis of this assumption, but it seems difficult to imagine why interbreeding between Neanderthal men and modern human females resulting in the incorporation of Neanderthal genes would have been more frequent than the reverse situation.

Even though our model of interaction and competition between Neanderthals and modern humans may not entirely correspond to the reality, it captures two important historical aspects that were neglected in previous studies. The first one is the documented progressive spread of modern humans in Europe (see Figures 1 and 2), and the second is the local and progressive demographic growth of Paleolithic populations, with density-dependent interactions with Neanderthals. The incorporation of these additional sources of realism cannot be handled by current analytical models, but it can be readily integrated into a coalescent simulation framework, showing that it will be possible in the future to predict patterns of molecular diversity among populations or species belonging to a particular ecological network. Given the long period of cohabitation of the two populations in Europe and ample opportunities to interbreed, the absence or extremely low number of admixture events between Neanderthals and modern humans is best explained by intersterility or reduced fitness of hybrid individuals, promoting these populations to the status of different biological species. No interbreeding between the two populations also strongly argues in favor of a complete replacement of previous members of the genus *Homo* by modern humans and against a multiregional evolution of H. sapiens (Eckhardt et al. 1993; Wolpoff et al. 2000). It thus gives more credit to the RAO hypothesis (Excoffier 2002; Stringer 2002), since some very divergent H. erectus mitochondrial sequences should have also been observed if interbreeding had occurred during the colonization of Eurasia by modern humans from Africa.

Our conclusions about the genetic incompatibility between modern humans and Neanderthals would however be wrong if the absence of Neanderthal mtDNA genes in the current gene pool of modern Europeans was due to some processes that were not incorporated into our model. For instance, a range expansion of Neolithic populations without genetic contacts with Paleolithic could have erased both Paleolithic and remaining Neanderthal genes, but as discussed above, there are evidences for a substantial contribution of Paleolithic populations to the current gene pool (Barbujani and Dupanloup 2002; Chikhi et al. 2002; Dupanloup et al. 2004), invalidating this theory. Also an extremely rapid range expansion of a very large and unsubdivided modern population would also be compatible with an absence of Neanderthal genes despite considerable admixture, like in the scenario shown in Figure 1A (Nordborg 1998; Serre et al. 2004), but the long duration of the replacement process would be difficult to justify in that case. Finally, the occurrence of a cultural or ecological barrier, and not necessarily of a genetic barrier, could have prevented the realization of biologically possible hybridizations. Under this scenario, Neanderthals and early modern humans would have just avoided each other, which is contradicted by the observation of technological exchanges between Neanderthals and Cro-Magnons (e.g., Hublin et al. 1996). Moreover, the fact that the two populations had a very similar economy (Klein 1999, p. 530), indicates they had occupied an overlapping ecological niche and had thus ample opportunities to meet. It therefore seems that our model of subdivided population and progressive range expansion, implying local contacts, competition, and potential hybridization is quite plausible. One of its merits is also to explain both the replacement of Neanderthals by modern humans through a better exploitation of local resources, but also the late colonization of Europe by modern humans, which would have been possible only after the emergence of refined Upper Paleolithic technologies giving a competitive edge over Neanderthal industries (Klein 1999, pp. 511–524).

## Materials and Methods

### 

#### Digital map of Europe

The simulated region corresponds to the geographical region encompassing Europe, the Near East and North Africa. It has been modeled as a collection of 7,500 square cells of 2,500 km^2^ each, arranged on a two-dimensional grid, with contours delimited by seas and oceans. Each cell harbors two demes, one potentially occupied by modern humans (HS) and one potentially occupied by Neanderthals (HN). Given the estimated range distribution of Neanderthals (Klein 2003), HN demes were allowed in only 3,500 cells, mainly located in the lower part of Europe and in the Near East (see Figure 2A). Three land bridges have been artificially added to allow the settlement of Great Britain and Sicily.

#### Simulation of the colonization of Europe by modern humans

The simulation of the colonization process in Europe is an extension of that described in absence of competition in a homogeneous square world (Ray et al. 2003). At the beginning of the simulation, 1,600 generations ago (corresponding to 40,000 y ago when assuming a generation time of 25 y), the HN demes are all filled at their carrying capacity, *K_HN_,* and, in the basic scenario, the population HS is assumed to be restricted to a single deme in the Near East at a position corresponding approximately to the present border between Saudi Arabia and Jordan. Note that alternative locations and a more widespread distribution are also envisioned in other scenarios (see Table 1).This source for the spatial and demographic expansion of modern humans into Europe has been chosen arbitrarily, as its exact origin is still debated (Bocquet-Appel and Demars 2000a; Kozlowski and Otte 2000). Since we model the evolution of mtDNA, we only simulate the spread of females, but we implicitly assume that there are the same number of males and females in each deme. The source deme for HS is assumed to be at its carrying capacity *K_HS_* of 40 females, corresponding to a density of about 0.06–0.1 individuals per km^2^ (including males and juveniles), in agreement with density estimates for Pleistocene hunter-gatherers (Steele et al. 1998; Bocquet-Appel and Demars 2000b). HS individuals can then migrate freely to each of the four neighboring HS demes at rate *m*/4. When one or more HS individuals enter an empty deme, it results in a colonization event, which initiates a local logistic growth process, with intrinsic rate of growth *r_HS_* per generation, and with limiting carrying capacity *K_HS_.* Interactions between the HS and the HN demes of the same cell are described below in more detail, and its combination with migrations between HS demes results in a wave of advance progressing from the Near East toward Europe and North Africa.

#### Demographic model incorporating competition and admixture

We describe here a demographic model of interaction between populations, incorporating competition and interbreeding between individuals of the HN and HS populations, as well as migration between neighboring demes from the same subdivided population. We distinguish here migrations events between HN and HS populations from migrations between neighboring HN or HS populations. We model the former ones as admixture events, whereas the latter ones correspond to true dispersal events. The life cycle of a population at a given generation is as follows: admixture, logistic regulation incorporating competition, followed by migration. This life cycle thus assumes that migration is at the adult stage. In line with previous work (Barbujani et al. 1995), the frequency of admixture events is assumed to be density-dependent. Within a given deme, each of the N_*i*_ individuals from the i-th population has a probability



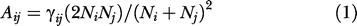



to reproduce successfully with one of the N_*j*_ members of the j-th population, and *γ*_*ij*_ represents the probability that such a mating results in a fertile offspring. Alternatively, *γ*_*ij*_ could represent the relative fitness of hybrid individuals or an index of disassortative mating. Following admixture, population densities are then first updated as



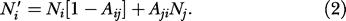



Our model of density regulation incorporating competition is based on the Lotka–Volterra interspecific competition model, which is an extension of the logistic growth model (Volterra 1926; Lotka 1932). For each population, a new density *N* ^″^_i_ is calculated from the former density as



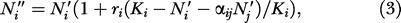



where *r_i_* is the intrinsic growth rate of the *i*-th population, *K_i_* is its carrying capacity, and *α_ij_* is an asymmetric competition coefficient (Begon et al. 1996, pp. 274–278). An *α_ij_* value of 1 implies that individuals of the *j*-th population have as much influence on those of population *i* as on their own conspecific, or that competition between populations is as strong as competition within a population. Lower values of *α_ij_* indicate lower levels of competition between populations than within populations; a value of zero implies no competition between individuals from different populations. We have decided here not to fix *α_ij_* values, but to make them density-dependent as



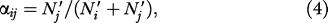



reflecting the fact that the influence of the members of a population on the other grows with its density. An example of the demographic transition between HN and HS is shown in Figure S2, together with the amount of admixture between the two populations. In the migration phase, each population of each deme can send emigrants to the same population in neighboring demes at rate *m.* *N* ^″^_i_ *m* emigrants are thus sent outward each generation, and distributed equally among the four neighboring demes, as described previously (Ray et al. 2003). If a gene is sent to an occupied deme, the migration event results in gene flow; otherwise, it results in the colonization of a new deme. This latter possibility only exists for the population of modern humans, since we assume that Europe was already fully colonized by Neanderthals. Finally, the densities of the two populations are updated as a balance between logistic growth, migration, and admixture as



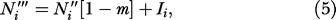



where *I_i_* is the number of immigrants received from neighboring demes.

#### Parameter calibration

We have calibrated the parameters of our simulation model from available paleodemographic information and from the estimated colonization time of Europe by modern humans. Estimates of the total number of hunter-gatherers living before Neolithic times range between 5 and 10 million (Coale 1974; Hassan 1981; Weiss 1984; Landers 1992; Chikhi et al. 2002), of whom about 1 million individuals were living in Europe. Taking a carrying capacity *K_HS_* of 40 females would imply the presence of 220,000 effective mtDNA genes in the 5,500 demes occupied by modern humans in Europe and the Middle East. Since this number represents only females, the total number of individuals living over Europe was multiplied by four to include men and juveniles, leading to a total density of about 880,000 HS individuals. This value of *K_HS_* corresponds to a density of 0.064 individuals per square kilometer, which is close to the value (0.04) used by some previous simulation of modern humans (Rendine et al. 1986; Barbujani et al. 1995) and well within the range obtained from actual hunter-gatherer groups (0.01–0.35; Binford 2001) or that estimated for ancient hunter-gatherers (0.015–0.2; Steele et al. 1998; Bocquet-Appel and Demars 2000b). The time required for the colonization of Europe by modern humans is the other information that was used to calibrate the growth rates, *r_HS_,* the rate of migration, *m_HS_*, and the Neanderthal carrying capacity *(K_HN_),* as these three parameters have an influence on the speed of the migration wave (Fisher 1937; Skellam 1951). Since modern humans arrived in Europe approximately 40,000 y ago and occupied the whole continent by 27,500 before present (BP) (Bocquet-Appel and Demars 2000b), the colonization process lasted approximately 500 generations, assuming an average generation time of 25 to 30 y (Tremblay and Vezina 2000; Helgason et al. 2003 ).

#### Scenarios of modern human range expansion in Europe

Among the many sets of parameter values leading to the appropriate colonization time and the complete disappearance of Neanderthals, we have retained the following scenarios. Scenario A: Origin of HS in a single deme of the Near East at the border between Saudi Arabia and Jordan, *m_HS_* = *m_HN_* = 0.25, *r_HN_* = 0.4, and *K_HN_* = 10, *r_HS_* = 0.4, *K_HS_* = 40. Note that a value of *K_HN_* of ten corresponds to a total density of about 140,000 Neanderthals over Europe (0.016 individuals per km^2^), which is of the same order of magnitude as the rare available estimates (250,000 Neanderthals, Biraben 2003). Under this scenario, we have only considered admixture events between HN females and HS males, such that γ_*HS,HN*_ = 0 . Eight alternative scenarios have been considered by using extreme values of the parameters of the model (*m, r, K,* European colonization time, place, and size of initial HS population). Scenario B is identical to scenario A, except that the HS origin is located in Iran. Scenario C uses the same parameters as scenario A, but the HS source is more diffuse and corresponds to a subdivided population of 25 demes (1,000 breeding females) surrounding the source deme defined in scenario A. Scenario D is identical to A, except that the initial HS population is even much more numerous (14,000 breeding females located in 1,400 demes) and occupies all the south area of the HN occupation zone. Scenario E is identical to A, but *r_HS_* is here equal to 0.8, which is the maximum growth rate estimated for the Paleolithic human population (Ammerman and Cavalli-Sforza 1984; Young and Bettinger 1995). Scenario F is identical to A, except that *m_HS_* is here much higher and equal to 0.5, implying that 50% of the women are recruited in adjacent demes. The carrying capacity of Neanderthals *K_HN_* had to be readjusted for scenarios E and F, which may appear as extreme, in order to maintain a colonization time of about 500 generations. It was indeed set to 25, giving a total density of HN of 350,000 individuals over Europe. Scenario G is identical to A, except that *r_HS_* is here equal to 0.6 and *m_HS_* is equal to 0.35, leading to a shorter colonization time of the European continent by HS. Under scenario G, the colonization time of Europe is approximately 8,000 y, which would correspond to the minimum colonization time estimated from direct fossil evidence, since the first European HS fossil is dated to about 36,000 y BP (Trinkaus et al. 2003), and the latest HN is dated around 28,000 y BP (Smith et al. 1999). Scenario H is identical to A, but admixture can occur between HN males and HS females as well, such that γ_*HS,HN*_ = γ_*HN,HS*_ . Finally, scenario I uses the same parameters as A, but a different demographic model. When a cell is colonized by HS, it is directly filled at *K_HS_* with an initial proportion γ of Neanderthals. Admixture thus occurs when demographic equilibrium is already reached, and not during the demographic growth as in the other models.

While the γ values are the true parameters of our model, they may not be very telling per se, and we have therefore chosen to quantify levels of interbreeding between populations using another parameterization, which is the average number of admixture events per deme between modern humans and Neanderthals. By performing a large series of simulations, we could find the values of *γ* leading to a given average number of admixture events per deme (e.g., 1/500, 1/100, 1/10, 1, 2, etc.). For instance, a value of 1/10 means that one admixture event occurred on average in one deme out of ten during the whole cohabitation period between HN and HS.

#### Coalescent simulations

For each scenario and for different interbreeding values, *γ_ij_,* the demography of the more than 14,000 demes is thus simulated for 1,600 generations. The density of all demes, the number of migrants exchanged between demes from the same population, and the number of admixture events resulting in gene movements between Neanderthals and modern humans are recorded in a database. This demographic database is then used to simulate the genealogy of samples of 40 genes drawn from 100 demes, representing a total of 4,000 modern human genes distributed over all Europe and corresponding approximately to the current sampling effort of European mtDNA sequence (Richards et al. 1996; Handt et al. 1998). The coalescent simulations proceed as described previously (Ray et al. 2003; Currat et al. 2004). The average proportion of sampled genes whose ancestors can be traced to some Neanderthal lineages was then computed over 10,000 simulations. The likelihood of each interbreeding coefficient, *γ_ij_,* is estimated for the different scenarios by the proportion of 10,000 simulations that lead to a Most Recent Common Ancestor of all 4,000 sampled mtDNA sequences being of modern human origin.

## Supporting Information

Figure S1 Proportion of Neanderthal Lineages in the European Population as a Function of the Average Number of Admixture Events per Deme between HN and HS These values are given for the nine scenarios (A–I) listed in Table 1, and for a new scenario A+Neol. This latter scenario is similar to A, except that the carrying capacity of the modern humans is increased by a factor 250 at the time of the Neolithic transition (320 generations BP). The influence of this demographic increase on the simulated HN proportion is very weak, as shown on this figure.
(357 KB TIF).

Figure S2 Evolution of the densities of demes HN (in black) and HS (in gray) within a cell simulated under demographic scenario A for *γ_ij_* = 0.4. The cell is colonized by HS at time −1520 ( 0 = present). The thin black line with white circles represents the distribution of admixture events, whose numbers are reported on the right axis (322 KB TIF).

## Abbreviations

BP: before present
HN: *Homo sapiens neanderthalensis*
HS: *Homo sapiens sapiens*
mtDNA: mitochondrial DNA
RAO: Recent African Origin Model
