# Supplementary figures and images for: Modern Humans Did Not Admix with Neanderthals during Their Range Expansion into Europe
Source: PLoS Biol. 2004 Nov 30;2(12):e421. doi: 10.1371/journal.pbio.0020421 (PMC532389; doi:10.1371/journal.pbio.0020421)

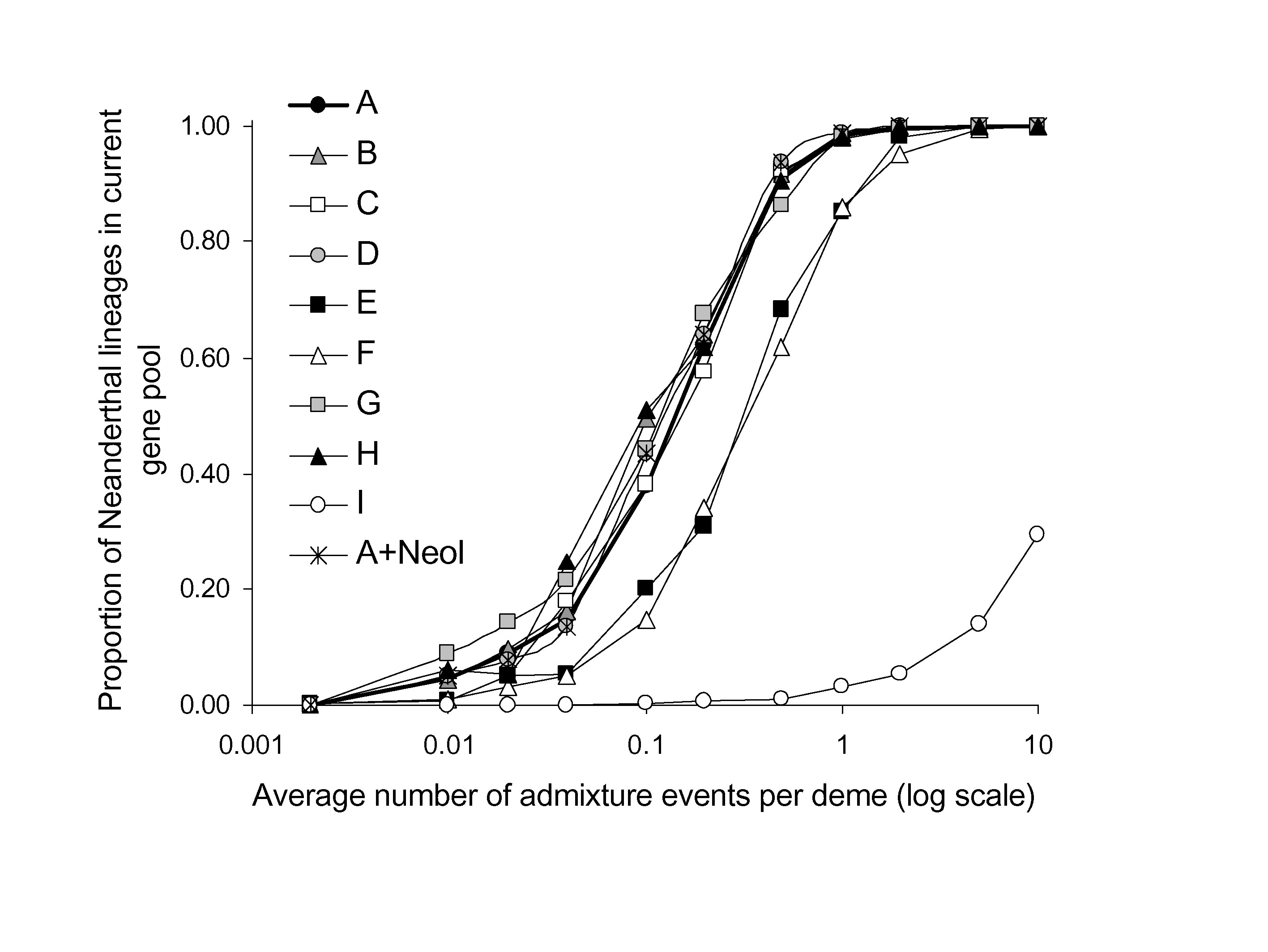

Supplement: Figure S1 — These values are given for the nine scenarios (A–I) listed in Table 1, and for a new scenario A+Neol. This latter scenario is similar to A, except that the carrying capacity of the modern humans is increased by a factor 250 at the time of the Neolithic transition (320 generations BP). The influence of this demographic increase on the simulated HN proportion is very weak, as shown on this figure. (357 KB TIF). [file pbio.0020421.sg001.tif]

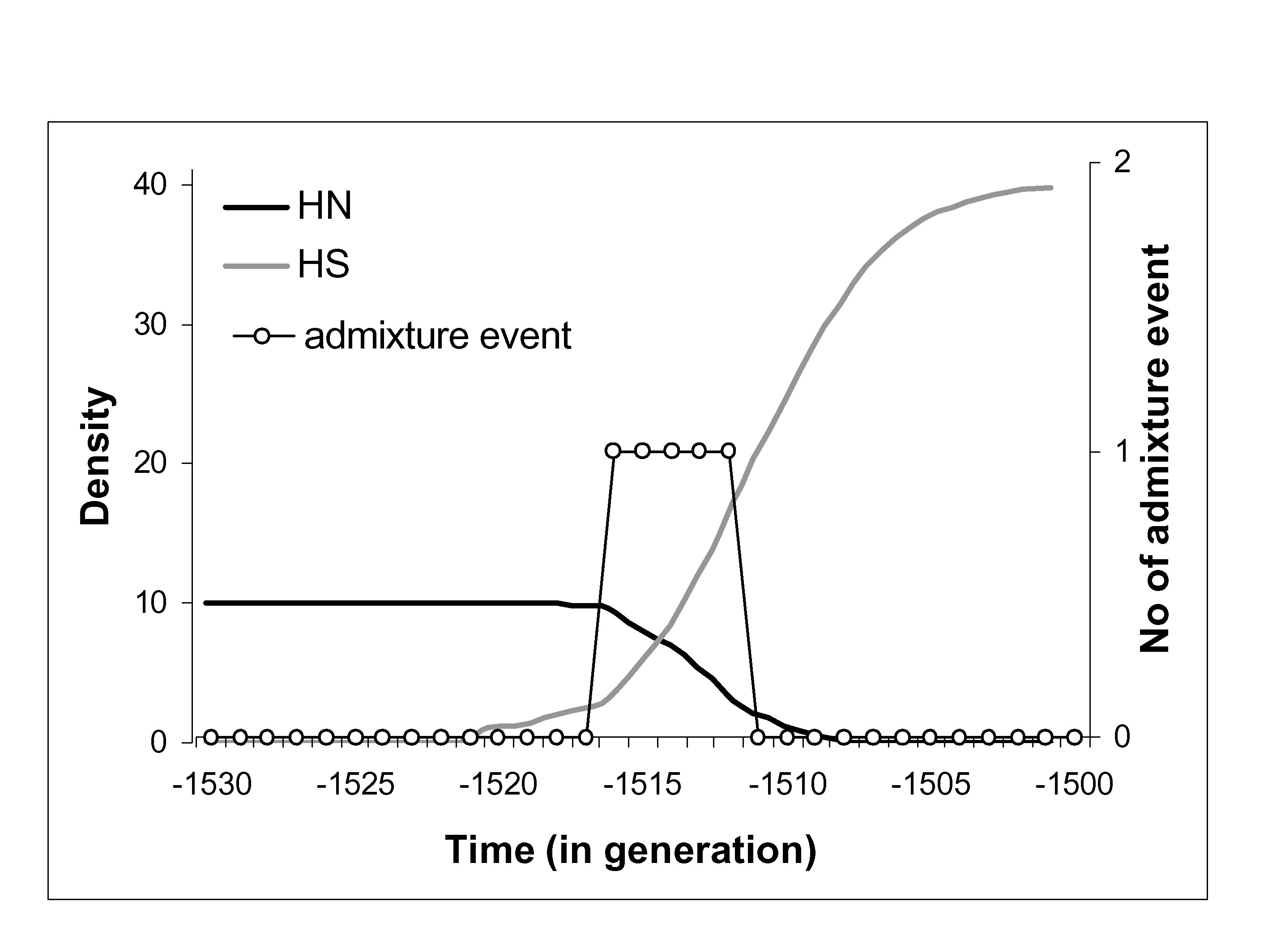

Supplement: Figure S2 — (322 KB TIF). [file pbio.0020421.sg002.tif]
